# AMPK Is Involved in Regulating the Utilization of Carbon Sources, Conidiation, Pathogenicity, and Stress Response of the Nematode-Trapping Fungus Arthrobotrys oligospora

**DOI:** 10.1128/spectrum.02225-22

**Published:** 2022-08-02

**Authors:** Wenjie Wang, Yining Zhao, Na Bai, Ke-Qin Zhang, Jinkui Yang

**Affiliations:** a State Key Laboratory for Conservation and Utilization of Bio-Resources, Key Laboratory for Microbial Resources of the Ministry of Education, School of Life Sciences, Yunnan University, Kunming, People’s Republic of China; University of Molise

**Keywords:** *Arthrobotrys oligospora*, AMP-activated protein kinase (AMPK), utilization of carbon sources, conidiation, trap formation

## Abstract

AMP-activated protein kinase (AMPK), a heterotrimeric complex, can sense energy and nutritional status in eukaryotic cells, thereby participating in the regulation of multiple cellular processes. In this study, we characterized the function of the catalytic α-subunit (SNF1) and the two regulatory β- and γ-subunits (GAL83 and SNF4) of AMPK in a representative nematode-trapping fungus, Arthrobotrys oligospora, by gene knockout, phenotypic analysis, and RNA sequencing. The ability of the AMPK complex mutants (including Δ*Aosnf1*, Δ*Aogal83*, and Δ*Aosnf4*) to utilize a nonfermentable carbon source (glycerol) was reduced, and the spore yields and trap formation were remarkably decreased. Moreover, AMPK plays an important role in regulating stress response and nematode predation efficiency. Transcriptomic profiling between the wild-type strain and Δ*Aosnf1* showed that differentially expressed genes were enriched for peroxisome, endocytosis, fatty acid degradation, and multilipid metabolism (sphingolipid, ether lipid, glycerolipid, and glycerophospholipid). Meanwhile, a reduced lipid droplet accumulation in Δ*Aosnf1*, Δ*Aogal83*, and Δ*Aosnf4* mutants was observed, and more vacuoles appeared in the mycelia of the Δ*Aosnf1* mutant. These results highlight the important regulatory role of AMPK in the utilization of carbon sources and lipid metabolism, as well as providing novel insights into the regulatory mechanisms of the mycelia development, conidiation, and trap formation of nematode-trapping (NT) fungi.

**IMPORTANCE** NT fungi are widely distributed in various ecosystems and are important factors in the control of nematode populations in nature; their trophic mycelia can form unique infectious devices (traps) for capturing nematodes. Arthrobotrys oligospora is a representative NT fungi which can develop complex three-dimensional networks (adhesive networks) for nematode predation. Here, we demonstrated that AMPK plays an important role in the glycerol utilization, conidiation, trap formation, and nematode predation of A. oligospora, which was further confirmed by transcriptomic analysis of the wild-type and mutant strains. In particular, our analysis indicated that AMPK is required for lipid metabolism, which is primarily associated with energy regulation and is essential for trap formation. Therefore, this study extends the functional study of AMPK in NT fungi and helps to elucidate the molecular mechanism of the regulation of trap development, as well as laying the foundation for the development of efficient nematode biocontrol agents.

## INTRODUCTION

The SNF1/AMP-activated protein kinase (AMPK) is a phylogenetically conserved kinase which can maintain cellular energy homeostasis in almost all eukaryotic cells (1, 2). The cell activates AMPK to actively phosphorylate downstream targets, and it acts to restore energy balance by initiating ATP synthesis pathways while shutting down other nonessential biosynthesis processes that consume ATP when the cellular energy is inadequate to meet the requirements of various physiological activities (3, 4). Furthermore, it has been shown that AMPK is structurally similar in most eukaryotes and is made up of three different subunits, including a catalytic α-subunit and two regulatory β- and γ-subunits (1). In mammals, AMPK is activated primarily by phosphorylation at Thr-172, which lies in the α-subunit, where phosphorylation is regulated by AMP or ADP (5, 6). However, it is not directly regulated by AMP in plants and fungi (7).

In Saccharomyces cerevisiae, the AMPK complex is composed of α-subunit SNF1; three alternate β-subunits, GAL83, SIP1, and SIP2; and γ-subunit SNF4 (8). Studies have shown that the SNF1 pathway of S. cerevisiae is involved in cell cycle regulation, cell proliferation, endocytosis, carbon source utilization, and stress (9). Moreover, AMPK plays a central role in the regulation of virulence, cell growth, and proliferation in several filamentous fungi. In plant-pathogenic fungus Magnaporthe oryzae, the AMPK complex consists of a catalytic subunit, SNF1, and two regulatory subunits SIP2 and SNF4, and it is crucial to aerial hyphae development, conidiogenesis, appressorial formation, and morphology. In addition, the defects of Δ*Mosnf1* were more serious than those of Δ*Mosip2* and Δ*Mosnf4* mutants. Interestingly, these three mutants inoculated on media with nonfermentable carbons as the sole carbon source displayed a significant reduction in hyphal growth (10). Similarly, deletion of the three genes encoding AMPK subunits in Fusarium graminearum resulted in defects in virulence, sexual development, and vegetative growth, whereas Δ*Fgsnf1* showed considerable vegetative growth defects compared to the Δ*Fggal83* and Δ*Fgsnf4* mutants (11). In Alternaria alternata, deletion of *Aasnf1* reduced the number of conidia, mycelial growth rates, pathogenicity, and utilization of carbon sources, while it significantly increased tolerance to cell wall-perturbing agents (12). Taken together, AMPK is closely involved in the regulation of growth and pathogenicity of diverse fungi.

Of particular interest to nematode-trapping (NT) fungi are the special trapping devices (traps) involved in capturing and killing nematodes (13–16). The traps are specifically formed by the vegetative hyphae of NT fungi, divided into adhesive knobs and columns, adhesive networks, and constricting and nonconstricting rings according to their different predatory methods (17–19). Arthrobotrys oligospora is one of the best-known species of NT fungi, which can develop adhesive networks for nematode predation, and its whole genome has previously been sequenced (20). Recently, some signaling proteins were identified to be involved in the mycelium development and pathogenicity of A. oligospora, including heterotrimeric G proteins (21–23), small GTPases (24, 25), and mitogen-activated protein kinase (MAPK) (26–28). The A. oligospora AMPK is composed of a catalytic α-subunit (AoSNF1) and two regulatory β- (AoGAL83) and γ-subunits (AoSNF4) according to the amino acid sequence alignment of orthologous AMPK subunits in S. cerevisiae. It has been reported that AMPK participates in the regulation of mycelial growth, sporulation, and pathogenicity in several pathogenic fungi, whereas its function remains unclear in NT fungi. In this study, we characterized the A. oligospora AMPK by targeted deletions and multiphenotypic analysis of the three AMPK complex subunits, and we performed transcriptomic analysis between the wild-type (WT) and mutant strains to further probe the regulatory mechanism of AoSNF1 in mycelium development, cellular processes, and trap formation of A. oligospora.

## RESULTS

### Bioinformatics analysis of the AMPK complex in A. oligospora.

**FIG 1:**
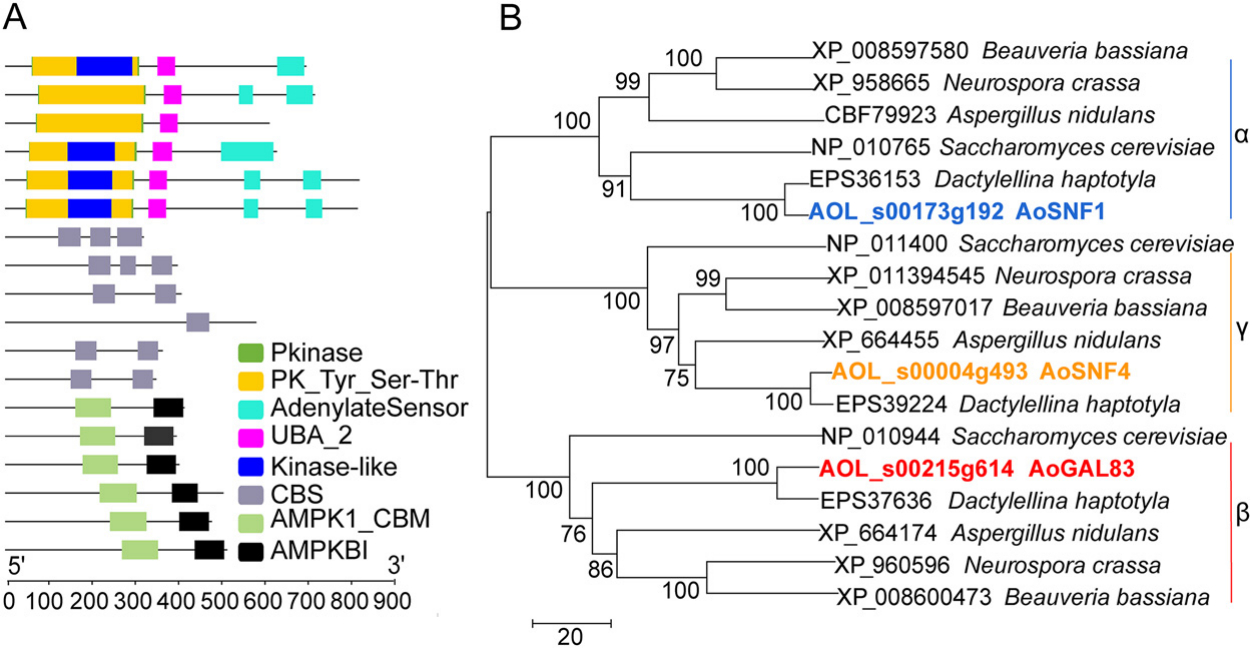
Functional domains and phylogenetic analysis of AMPK subunits in different fungi. The homologous sequences of the A. oligospora AMPK subunits in different fungi were retrieved from the NCBI database. The phylogenetic tree was constructed by MEGA 5.0 software using the neighbor-joining method. GenBank accession numbers precede the species names. (A) Conserved domains. Colored regions represent the conserved domains. (B) Phylogenetic tree. Α, alpha subunit; γ, gamma subunit; β, beta subunit.

AMPK is a heterotrimer, composed of a catalytic α-subunit and regulatory β- and γ-subunits. In the A. oligospora AMPK, the α-subunit AoSNF1 contains more amino acid (aa) residues (822 aa), a greater molecular weight (90.57 kDa), and more functional domains (protein kinase domain, SNF1, ubiquitin-associated domain, AMPK, C-terminal adenylate sensor domain) than the β-subunit AoGAL83 (399 aa; 43.26 kDa; ASC domain and AMPK glycogen-binding domain) and the γ-subunit AoSNF4 (366 aa, 40.93 kDa, CBS domain). Additionally, the conserved domains contained in the orthologs of the AMPK subunits from other fungi are similar to those of A. oligospora, whereas the α-subunit of Aspergillus nidulans has no C-terminal adenylate sensor domain, and only one CBS domain is detected in the γ-subunit (Fig. 1A). AoSNF1 also contains a Ser/Thr protein kinase active site (Interpro accession no. IPR008271) and a protein kinase ATP-binding site (Interpro accession no. IPR017441), whereas AoGAL83 and AoSNF4 do not. Phylogenetic analysis showed that the three AMPK subunits (α-, β-, and γ-subunits) from various fungi are divided into different branches (Fig. 1B).

### Deletion of AMPK impairs the utilization of carbon sources.

**FIG 2:**
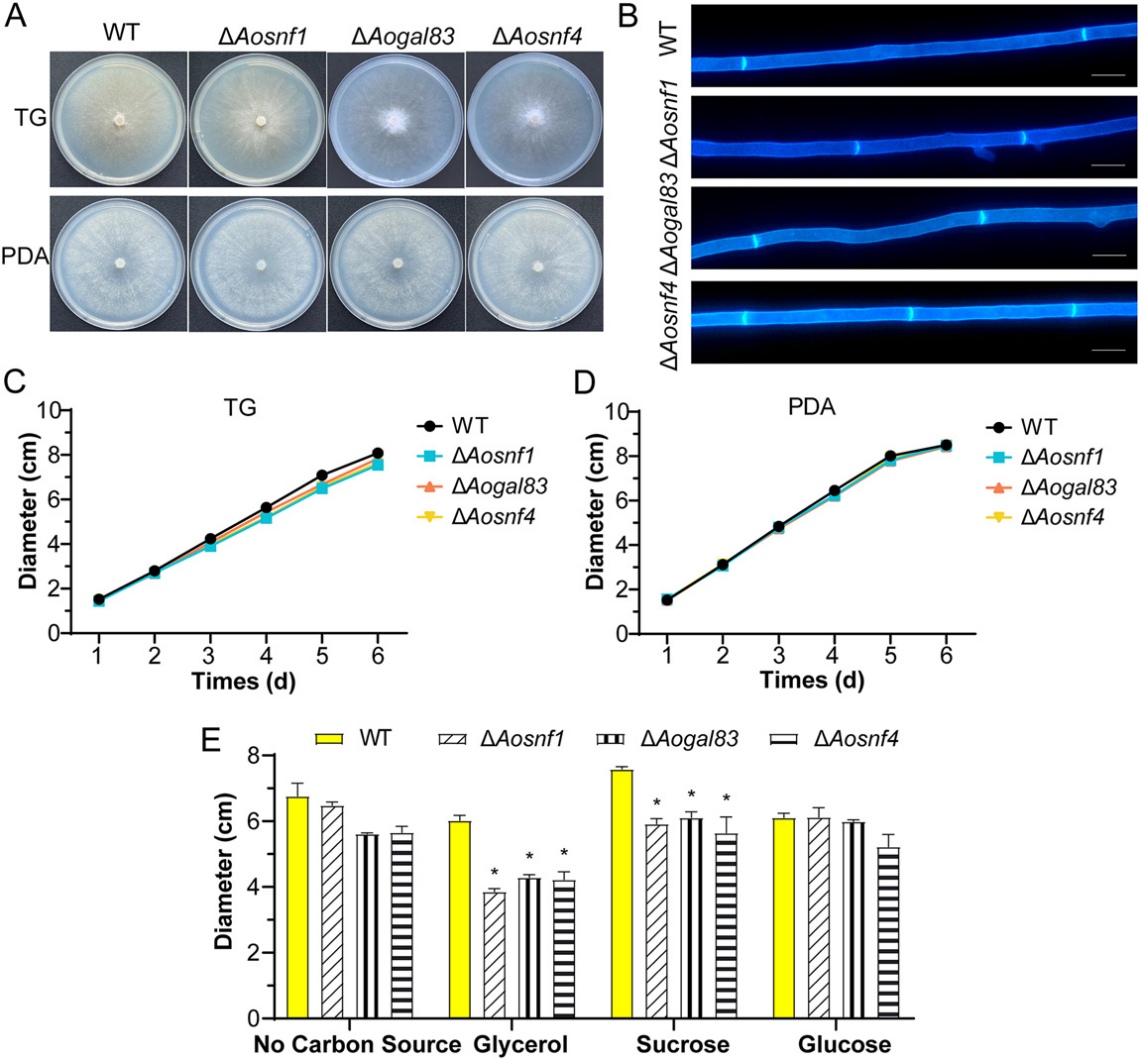
Colony morphology and diameters between the wild-type (WT) and AMPK complex mutant (Δ*Aosnf1*, Δ*Aogal83*, and Δ*Aosnf4*) strains. (A) Colonies of strains cultured on TG and PDA plates for 6 days at 28°C. (B) Mycelia of the WT and mutant strains on PDA. Mycelia were stained with calcofluor white (CFW). Scale bar, 5 μm. (C) Colony diameters of the WT and mutant strains cultured on TG plates for 6 days. (D) Mycelia of the WT and mutant strains on PDA. (E) Colony diameters of the WT and mutant strains cultured on CDA medium with different carbon sources for 6 days. *, significant difference between the mutant and WT strains (*P < *0.05).

Three positive transformations for *Aosnf1* and two transformations for genes *Aogal83* and *Aosnf4* were generated, respectively, as described in Materials and Methods, and they were confirmed by PCR and Southern blot analyses (see Fig. S1 in the supplemental material). Because the independent mutants of each gene exhibited similar phenotypic traits, a single mutant for each gene was selected to represent the results of the analysis. Compared with the WT strain, the hyphal growth and morphology of the Δ*Aosnf1*, Δ*Aogal83*, and Δ*Aosnf4* mutants almost displayed no difference on tryptone-glucose (TG) and potato dextrose agar (PDA) media (Fig. 2A to D), whereas there was a remarkable difference in colony diameter on Czapek-Dox agar (CDA) medium modified with different carbon sources (Fig. 2E). In addition, aerial hyphae were more abundant on PDA and TG medium (Fig. 2A), whereas the hyphae of the WT and mutant strains on CDA medium were very sparse, and almost no aerial mycelia could be observed.

The colony diameters of Δ*Aosnf1*, Δ*Aogal83*, and Δ*Aosnf4* mutants were smaller on CDA medium without a carbon source than the wild-type (WT) strain, as well as on the CDA medium supplemented with glucose as the only carbon source. However, the colony diameters of the mutant strains were much smaller than those of the WT strain on the CDA medium supplemented with glycerol or sucrose as the carbon source. Specifically, when glycerol (nonfermentative carbon source) was used as the only carbon source, the colony diameters of the Δ*Aosnf1*, Δ*Aogal83*, and Δ*Aosnf4* mutants were decreased by 36%, 29%, and 30%, respectively (Fig. 2E).

### AMPK is required for conidiation.

**FIG 3:**
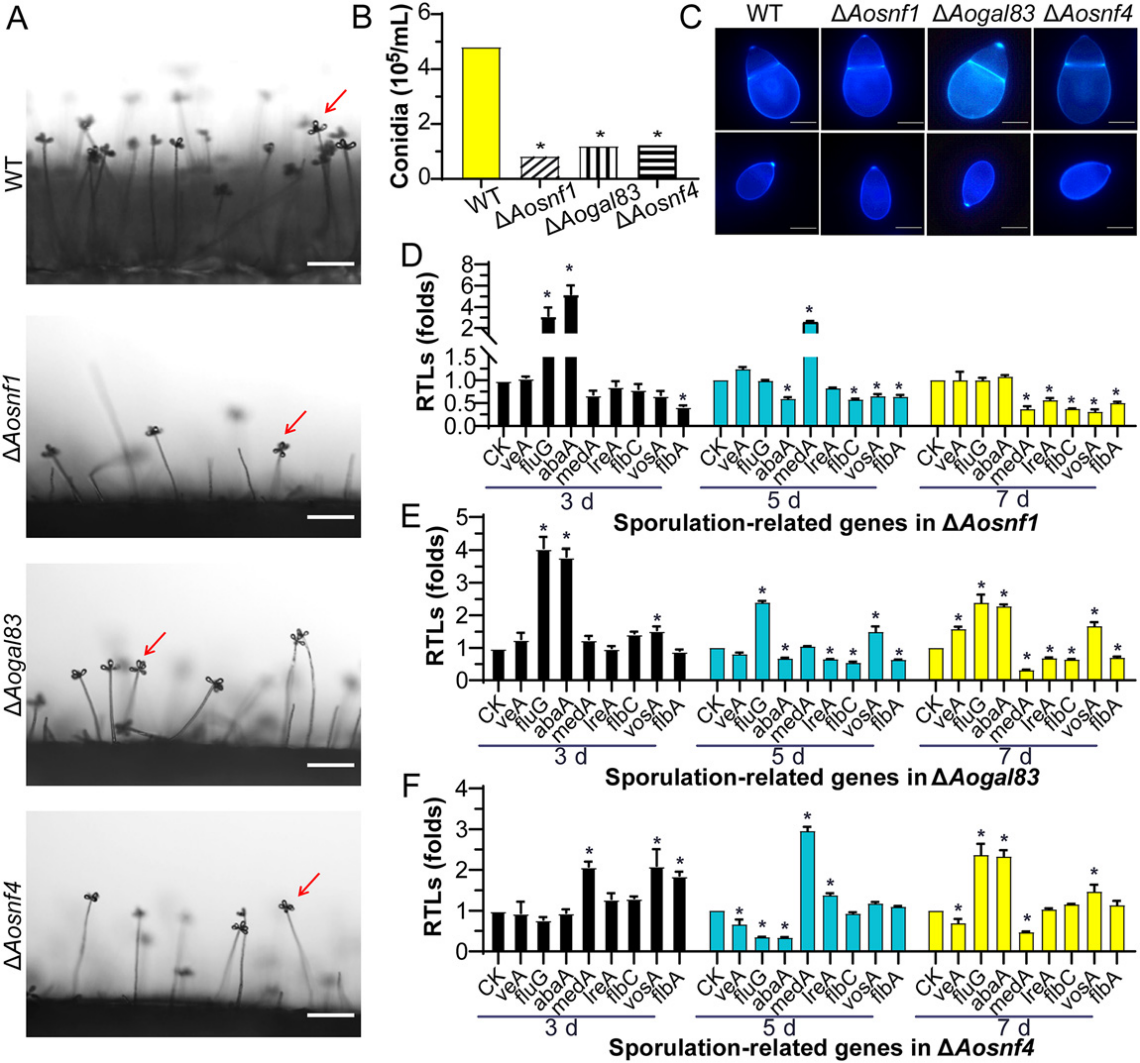
Conidiation and transcript levels of sporulation-related genes of the WT and mutant strains. (A) Sporulation of the WT and mutant strains on PDA medium. Red arrows, conidium. Scale bar, 50 μm. (B) Spore yields. (C) Conidia stained with calcofluor white (CFW) and examined under a confocal laser scanning microscope. Scale bar, 5 μm. (D to F) Relative transcription levels (RTLs) of sporulation-related genes in the WT and mutant strains. *, significant difference between the mutant and WT strains (*P < *0.05). CK was used as the standard (RTL = 1) for statistical analysis of the RTL of each gene under a given condition.

Deletion of *Aosnf1*, *Aogal83*, and *Aosnf4* resulted in a significant reduction (*P < *0.05) in spore production. Compared with the WT strain, the spore yields of Δ*Aosnf1*, Δ*Aogal83*, and Δ*Aosnf4* mutants were decreased by 81.5%, 74.7%, and 73.9%, respectively. Among them, the Δ*Aosnf1* mutant produced fewer spores than the WT and other two mutant strains (Fig. 3A and B). However, the spore morphology of all mutants showed no difference from the WT strain (Fig. 3C).

To further probe the function of AMPK in regulating conidiation, the transcript levels of eight sporulation-related genes (*veA*, *fluG*, *abaA*, *medA*, *lreA*, *flbC*, *vosA*, and *flbA*) (29) in the WT and mutant strains cultured at different growth stages were examined by reverse transcription-PCR (RT-PCR). In the Δ*Aosnf1* mutant, two genes (*fluG* and *abaA*) were upregulated and one gene (*flbA*) downregulated on the third day, one gene (*medA*) was upregulated and four genes (*abaA*, *flbC*, *vosA*, and *flbA*) downregulated on the fifth day, and five genes (*medA*, *lreA*, *flbC*, *vosA*, and *flbA*) were downregulated on the seventh day (Fig. 3D). In the Δ*Aogal83* mutant, three genes (*fluG*, *abaA*, and *vosA*) were upregulated on the third day, and two genes (*fluG* and *vosA*) were upregulated and four genes (*abaA*, *lreA*, *flbC*, and *flbA*) downregulated on the fifth day. On the seventh day, *veA*, *flbC*, *abaA*, and *vosA* genes were upregulated, and *medA*, *lreA*, *flbC*, and *flbA* genes were downregulated (Fig. 3E). In the Δ*Aosnf4* mutant, three genes (*medA*, *vosA*, and *flbA*) were upregulated on the third day, two genes (*medA* and *lreA*) were upregulated and three genes (*veA*, *fluG*, and *abaA*) downregulated on the fifth day, and three genes (*fluG*, *abaA*, and *vosA*) were upregulated, and two genes (*medA* and *veA*) were downregulated on the seventh day (Fig. 3F).

### AMPK plays a crucial role in trap formation and nematode predation.

**FIG 4:**
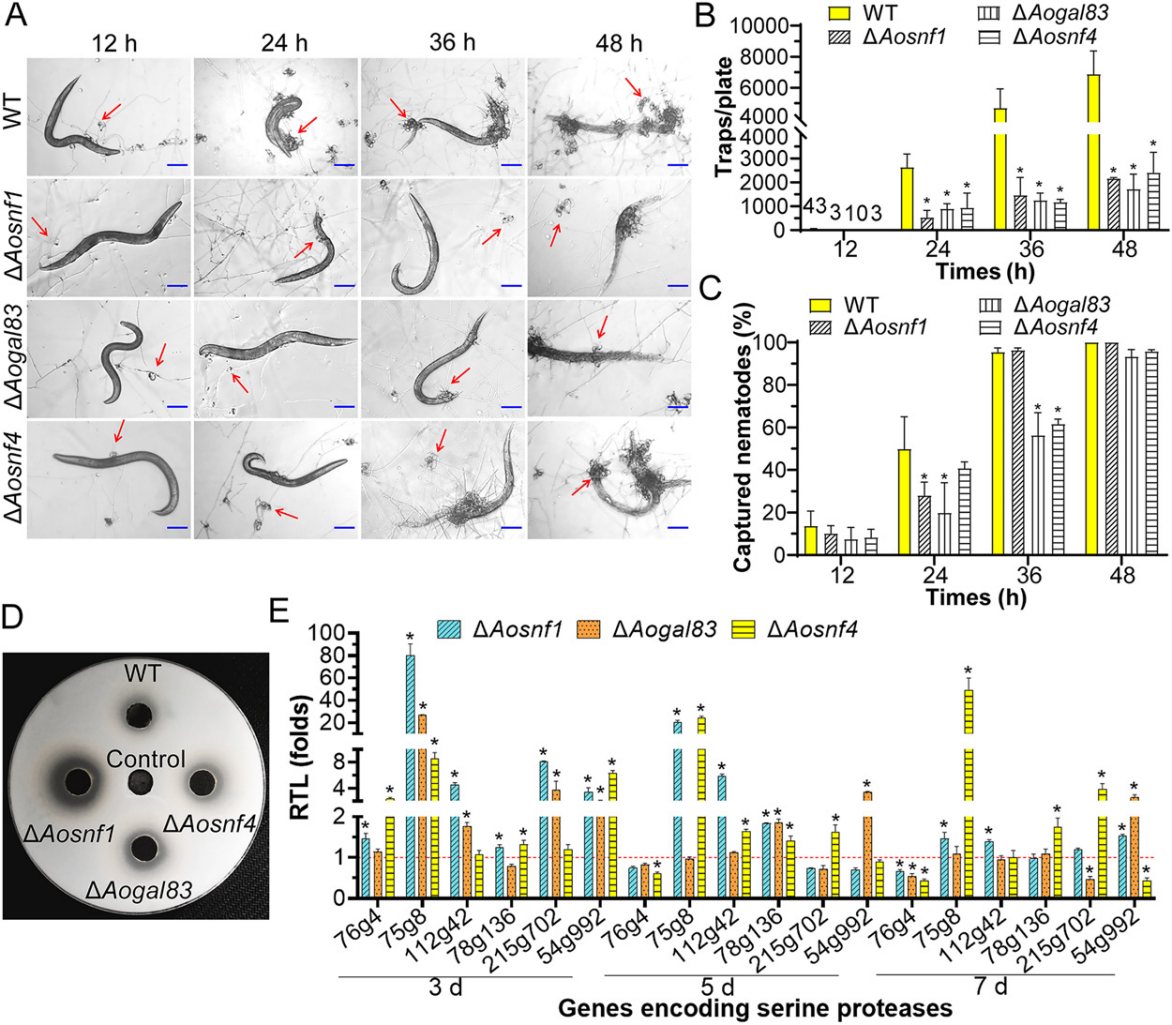
Trap formation, nematocidal activity, and extracellular proteolytic activity in the WT and mutant strains. (A) Traps induced with nematodes at 12 h, 24 h, 36 h, and 48 h. Red arrows, traps. Scale bar, 100 μm. (B) Comparison of traps produced by the WT and mutant strains at different time points. (C) Percentage of captured nematodes at different time points. (D) Comparison of extracellular protease activity. (E) Relative transcription levels (RTLs) of the genes encoding serine proteases in the WT and mutant strains cultured at different time points. The red line is the standard (RTL = 1) for statistical analysis of the RTL of each gene under a given condition. *, significant difference between the mutant and WT strains (Tukey’s HSD, *P < *0.05).

We observed significant differences (*P < *0.05) in producing traps between the WT and mutant strains after being induced with Caenorhabditis elegans (Fig. 4A). At 12 h, the WT and mutant (Δ*Aosnf1*, Δ*Aogal83*, and Δ*Aosnf4*) strains started to produce a few immature traps containing a half or single hyphal loop, and they formed an average of 43, 3, 10, and 3 traps per plate, respectively. All the WT and mutant strains produced a certain number of mature traps at 24 h, but the WT strain produced significantly (*P < *0.05) more traps than the three mutant strains, as well as at 36 and 48 h (Fig. 4B). At 12 h, fewer nematodes were captured by the mutants than by the WT strain. At 24 h, 50.0% of the nematodes were captured by the WT strain, whereas 28.1%, 19.9%, and 40.87% of the nematodes were captured by the Δ*Aosnf1*, Δ*Aogal83*, and Δ*Aosnf4* mutants, respectively. At 36 h, 95.4% of the nematodes were captured by the WT strain, whereas 96.4%, 56.3%, and 61.7% of the nematodes were captured by Δ*Aosnf1*, Δ*Aogal83*, and Δ*Aosnf4*, respectively. However, at 48 h, more than 95% of the nematodes were captured by the WT and mutant strains (Fig. 4C).

According to the size of hydrolytic circles on the milk plate, the Δ*Aosnf1* mutant strain produced more extracellular hydrolytic enzymes (proteases) than the WT strain, whereas the hydrolyzed circles of the Δ*Aogal83* and Δ*Aosnf4* mutants were almost the same as those of the WT strain (Fig. 4D). Furthermore, we determined the transcription of six genes (*76g4*, *75g8*, *112g42*, *78g136*, *215g702*, and *54g992*) encoding serine proteases by RT-PCR. Except for the *76g4* gene, which was upregulated on the third day and downregulated on the seventh day, other genes (*75g8*, *112g42*, *78g136*, *215g702*, and *54g992*) were remarkably upregulated to various degrees in the Δ*Aosnf1* mutant at different time points. In the Δ*Aogal83* mutant, four genes (*75g8*, *112g42*, *215g702*, and *54g992*) and two genes (*78g136* and *54g992*) were substantially upregulated on the third and fifth days, respectively. On the seventh day, one gene (*54g992*) was upregulated, and two genes (*76g4* and *215g702*) were downregulated. Furthermore, in the Δ*Aosnf4* mutant, four genes (*76g4*, *75g8*, *78g136*, and *54g992*), four genes (*75g8*, *112g42*, *78g136*, and *215g702*), and three genes (*75g8*, *78g136*, and *215g702*) were remarkably upregulated at the three different time points, whereas one gene (*76g4*) and two genes (*76g4* and *54g992*) were downregulated on the fifth and seventh days, respectively (Fig. 4E).

### AMPK regulates stress response.

**FIG 5:**
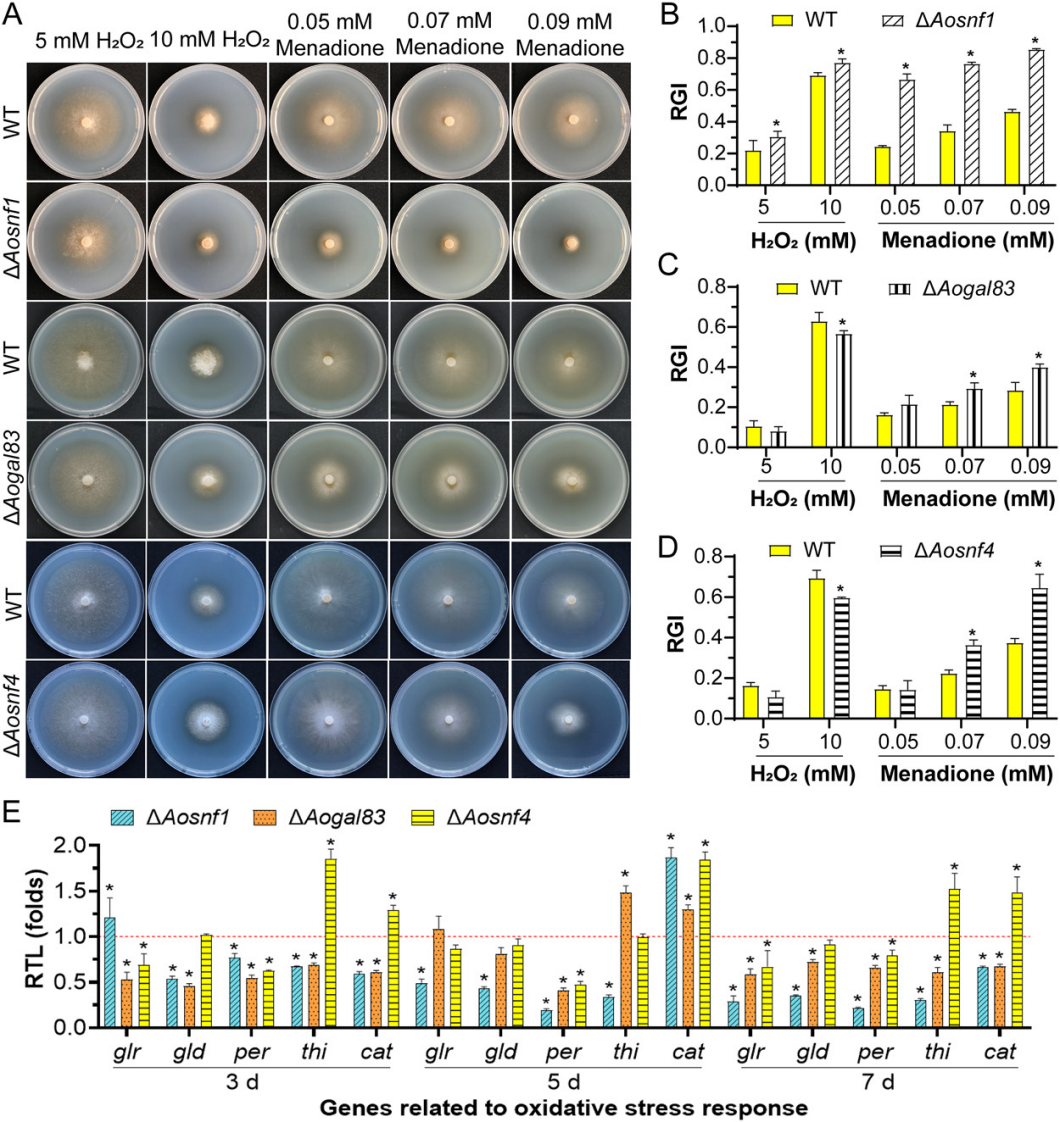
Stress response between the WT and mutant strains. (A) Growth of the WT and AMPK mutant strains on media supplemented with oxidants. (B to D) Relative growth inhibition (RGI) of the WT and mutant strains (Δ*Aosnf1*, Δ*Aogal83*, and Δ*Aosnf4*) cultured on TG plates supplemented with H_2_O_2_ (5 and 10 mM) and menadione (0.05, 0.07, and 0.09 mM). (E) RTLs of the genes related to oxidative stress response in the WT and mutants cultured at different time points. The red line indicates the standard (RTL = 1) for statistical analysis of the RTL of each gene under a given condition. *, significant difference between the mutant and WT strains (Tukey’s HSD, *P < *0.05).

Compared with the WT strain, the mycelial growth of the Δ*Aosnf1* mutant was more suppressed when cultured on TG plates supplemented with oxidative agents (H_2_O_2_ and menadione), while the Δ*Aogal83* and Δ*Aosnf4* mutants were suppressed under 0.07 and 0.09 mM menadione but grew faster under 10 mM H_2_O_2_ (Fig. 5A). The relative growth inhibition (RGI) values of the AMPK mutants were significantly increased (*P < *0.05) compared to the WT strain in the presence of 0.07 and 0.09 mM menadione, and the RGI value of the Δ*Aosnf1* mutant was also remarkably increased under 0.05 mM menadione, as well as 5 and 10 mM H_2_O_2_. In contrast, the RGI values were decreased in the Δ*Aogal83* and Δ*Aosnf4* mutants under 10 mM H_2_O_2_ (Fig. 5B to D). Meanwhile, the Δ*Aosnf1* mutant grew faster than the WT strain under 0.09 mg/mL Congo red and 0.2 M NaCl (Fig. S2A and B). The RGI value of the Δ*Aogal83* mutant was considerably decreased under 0.06 mg/mL Congo red and increased under 0.02% SDS, as well as 0.5 and 0.75 M sorbitol (Fig. S2C and D), whereas that of Δ*Aosnf4* was remarkably decreased under 0.06 and 0.09 mg/mL Congo red and increased under 0.3 M NaCl and 0.25 M sorbitol (Fig. S2E and F).

We further analyzed the expression of five genes (*glr*, *gld*, *per*, *thi*, *prx*, and *cat*) associated with oxidative stress response in the WT and mutants (Fig. 5E). In the Δ*Aosnf1* mutant, with the exception of the *glr* and *cat* genes, which were upregulated on the third and fifth days, respectively, the expression levels of the other genes were remarkably downregulated at different time points. In the Δ*Aogal83* mutant, the expression levels of all five genes were downregulated on the third and seventh days, one gene *per* was downregulated and two genes (*thi* and *cat*) upregulated on the fifth day. In the Δ*Aosnf4* mutant, two genes (*glr* and *per*) were downregulated and two genes (*thi* and *cat*) upregulated on the third and seventh days, respectively. Additionally, one gene (*cat*) was upregulated, and gene *per*r was downregulated on the fifth day.

### Transcriptomic insight into the regulatory role of AoSNF1.

The WT and Δ*Aosnf1* mutant strains were induced with nematodes for 0 and 12 h, and then the mycelial samples were collected for RNA sequencing. Quality control data showed that the error rate of transcriptome data was less than 0.1%, while the Phred-like quality scores of the samples at the Q20 and Q30 levels were both higher than 90%, and the GC content was 48.23 to 48.82% (Table S1). The quality assessment of the transcriptome showed that the saturation quality of the sequencing was high, the sequencing depth was sufficient to cover most expressed genes, and the sequences obtained by sequencing were evenly distributed on the genes. In the principal-component analysis (PCA), the proportions of PC1 and PC2 that could distinguish samples were 73.81% and 8.31%, respectively. Moreover, the four samples were distributed in different regions, indicating high similarity and good reproducibility of the three replicates (Fig. S3). To verify the transcriptome data, the transcription levels of 11 selected genes related to peroxisome, glycerolipid metabolism, and endocytosis were examined by RT-PCR. The results showed that expression levels of all six genes related to peroxisome were upregulated, whereas genes related to glycerolipid metabolism and endocytosis (except *210g5*) were downregulated at 0 and 12 h. These results were highly consistent with transcriptome analysis (Fig. S4).

**FIG 6:**
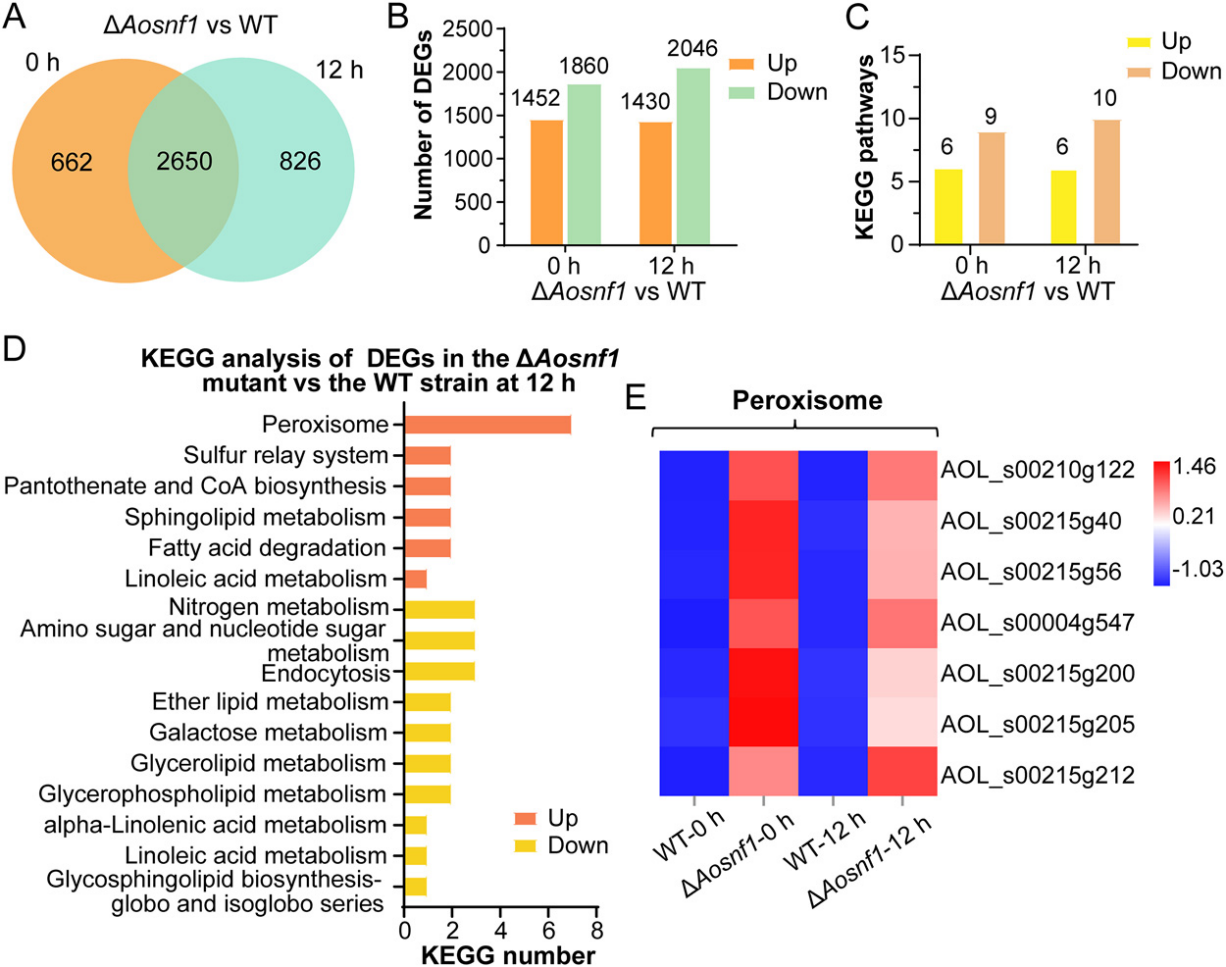
Comparison of differentially expressed genes (DEGs) between the Δ*Aosnf1* mutant and WT strains. (A) Venn analysis of DEGs. (B) Number of upregulated and downregulated DEGs in the Δ*Aosnf1* mutant versus the WT strain at 0 and 12 h. (C) Number of KEGG pathways obtained after KEGG analysis for each gene list. (D) KEGG analysis of upregulated and downregulated DEGs in the Δ*Aosnf1* mutant versus the WT strain at 12 h. (E) Heatmap showing the expression levels of DEGs involved in the peroxisome. Gene expression patterns are shown on a log_10_ scale. Red boxes, upregulated clusters; blue boxes, downregulated clusters.

There were 3,312 differentially expressed genes (DEGs) in the Δ*Aosnf1* mutant compared with the WT strain at 0 h, of which 662 were specifically expressed. At 12 h, 3,476 DEGs were found, of which 826 were specifically expressed (Fig. 6A). Among the total DEGs, 1,452 genes were upregulated, and 1,860 genes were downregulated at 0 h. At 12 h, 1,430 genes were upregulated, and 2,046 were downregulated (Fig. 6B). In gene ontology (GO) enrichment analysis, upregulated genes were enriched for 65 GO terms, and downregulated genes were enriched for 76 GO terms at 0 h, while upregulated genes were enriched for 93 GO terms, and downregulated genes were enriched for 89 GO terms at 12 h (Fig. S5A). The top 15 GO terms enriched by upregulated and downregulated DEGs at different time points almost all belonged to biological processes. The upregulated GO terms included coenzyme biosynthetic process, pantothenate biosynthetic process, and pantothenate metabolic process at 0 h and peroxisome, oxidoreductase activity, and oxidation-reduction process at 12 h (Fig. S5B and C). The downregulated GO terms included response to oxidative stress, nitrate assimilation, nitrate metabolic process, response to nutrient, and regulation of generation of precursor metabolites and energy at 0 h and nitrate assimilation, nitrate metabolic process, response to nutrient, NADPH activity, and oxidoreductase activity at 12 h (Fig. S5D and E).

In the Kyoto Encyclopedia of Genes and Genomes (KEGG) enrichment analysis, there were 15 and 16 enriched KEGG pathways at 0 and 12 h, respectively (Fig. 6C). Specifically, the KEGG pathways enriched for upregulated genes were the same at different time points, including peroxisome, sulfur relay system, pantothenate and CoA biosynthesis, sphingolipid metabolism, fatty acid degradation, and linoleic acid metabolism (Fig. 6D and Fig. S6). In addition, KEGG pathways enriched for downregulated genes were very similar at 0 and 12 h, including glycerophospholipid metabolism, galactose metabolism, glycerolipid metabolism, ether lipid metabolism, nitrogen metabolism, alpha-linolenic acid metabolism, linoleic acid metabolism, and glycosphingolipid biosynthesis (globo and isoglobo series), but not glyoxylate and dicarboxylate metabolism at 0 h and not endocytosis and amino sugar and nucleotide sugar metabolism at 12 h (Fig. 6D and Fig. S6). On the basis of the KEGG analysis, we performed cluster analysis of peroxisome-related genes that were enriched (Fig. 6E). The results showed that the expression levels of genes related to peroxisome in the Δ*Aosnf1* mutant were higher than those of the WT strain, and the gene upregulation at 0 h was more remarkable than that at 12 h. Of these, three genes (*4g547*, *215g40*, and *215g205*) were involved in peroxisome biosynthesis, and four genes (*215g56*, *215g212*, *210g122*, and *215g200*) were involved in fatty acid oxidation.

### AMPK plays a role in lipid metabolism and cellular components.

**FIG 7:**
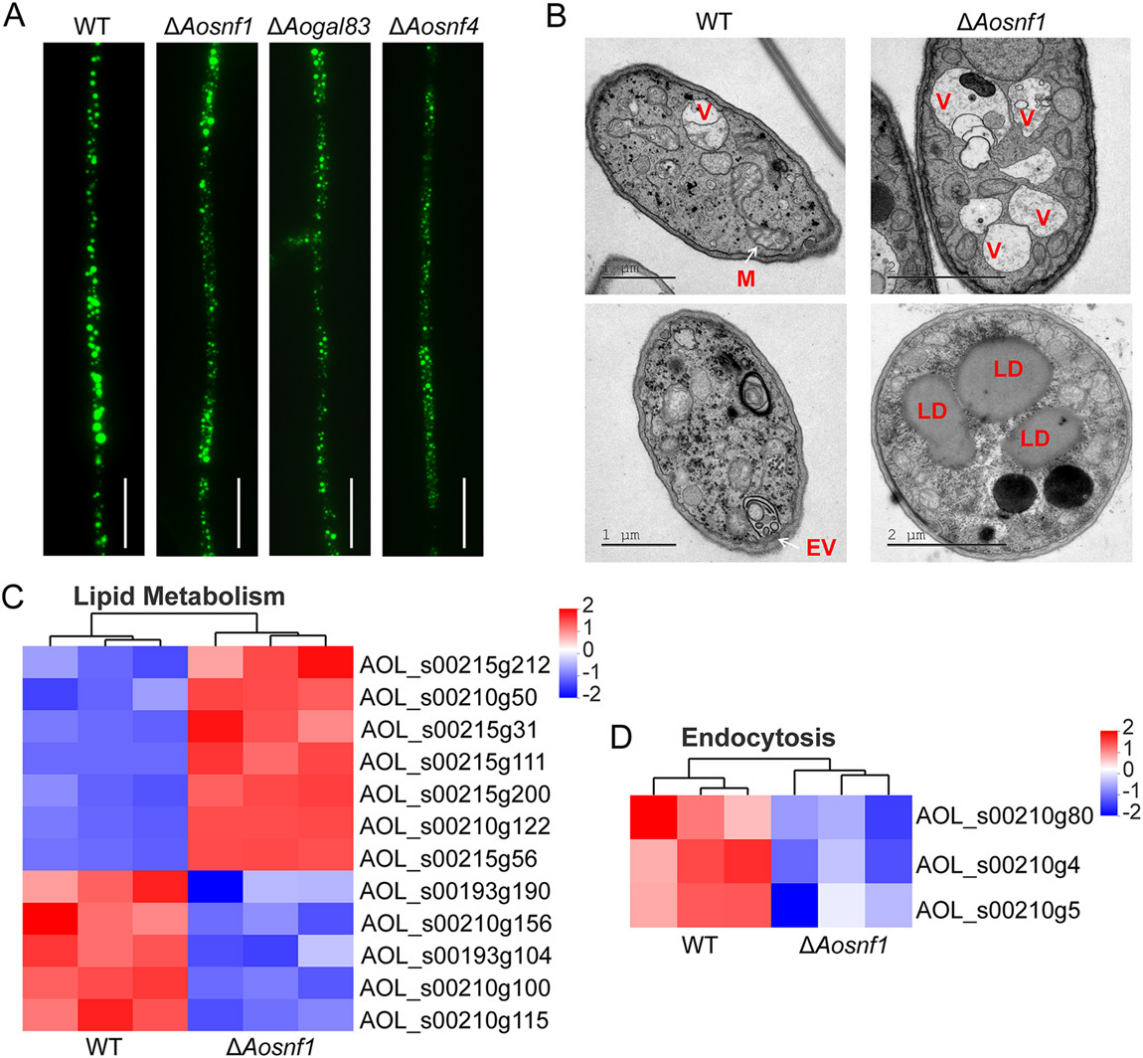
Lipid metabolism and cellular components in the WT and mutant strains. (A) Lipid droplets (LDs) observed in mycelia of the WT and mutant strains. Scale bar, 10 μm. (B) Cellular components were observed in the WT and Δ*Aosnf1* mutant strains by transmission electron microscopy. V, vacuole; M, mitochondrion; EV, endocytic vesicle; LD, lipid droplet. (C) Heatmap showing the genes involved in lipid metabolism. (D) Heatmap showing the genes involved in endocytosis. Gene expression patterns are shown on a log_10_ scale. Red boxes, upregulated clusters; blue boxes, downregulated clusters.

By staining the lipid droplets (LDs) in mycelia, the difference in LD volume between the WT and mutant strains was observed under a confocal laser scanning microscope. Compared with the WT strain, the volume of LDs was decreased in the Δ*Aosnf1*, Δ*Aogal83*, and Δ*Aosnf4* mutants (Fig. 7A). In addition, the transmission electron microscopy (TEM) results showed that dividing mitochondria and endocytic vesicles could be observed in the WT strain. In contrast, more vacuoles and lipid droplets (LDs) were observed in the Δ*Aosnf1* mutant (Fig. 7B). Meanwhile, the expression levels of genes involved in lipid metabolism and endocytosis were visualized using heatmaps (Fig. 7C and D). Compared to the WT strain, the expression levels of the gene encoding fatty acid oxygenase PpoC (AOL_s00215g31) and four genes (*215g212*, *215g56*, *210g122*, and *215g200*) involved in fatty acid β-oxidation were upregulated in the Δ*Aosnf1* mutant, whereas *193g190*, *210g156*, *193g104*, *210g100*, and *210g115* were downregulated (Fig. 7C). The expression levels of genes (*210g80*, *210g4*, and *210g5*) involved in endocytosis were downregulated in the Δ*Aosnf1* mutant, whereas they were upregulated in the WT strain (Fig. 7D).

## DISCUSSION

AMPK, also known as sucrose nonfermenting (SNF1) protein kinase, is a key cellular energy sensor (30). Previous studies showed that it is involved in the regulation of carbon source utilization, sporulation, pathogenicity, and stress response in some filamentous fungi and yeasts (9, 10, 12). In this study, we found that AMPK plays a conserved role in carbon source utilization, sporulation, and stress response, along with a crucial role in trap formation and lipid metabolism in NT fungus A. oligospora.

Compared with the WT strain, the mycelial growth of AMPK complex mutant strains (Δ*Aosnf1*, Δ*Aogal83*, and Δ*Aosnf4*) cultured on PDA and TG was not affected, but it was significantly affected on CDA medium with different carbon sources, especially when a nonfermentable carbon source (glycerol) was used as the only carbon source. In addition, the aerial hyphae of all strains on CDA medium were very sparse. In S. cerevisiae, SNF1 was involved in meiosis and invasive growth (8). Deletion of the *snf1* and *snf4* genes resulted in the mutants being unable to grow on fermentable carbon source sucrose and nonfermentable carbon source glycerol or ethanol (30–32). Similarly, in M. oryzae, the mycelia of Δ*Mosnf1*, Δ*Mosip2*, and Δ*Mosnf4* mutants grew normally, whereas growth was inhibited when inoculated on the medium with nonfermentable carbon as the sole carbon source (10). In A. alternata, the hyphal growth of the Δ*Aasnf1* mutant was defective, and the colony diameter and mycelia radial growth were decreased on minimal medium supplemented with glucose or glycerol (12). These findings showed that AMPK plays a varied role in the mycelial growth of A. oligospora and other fungi, with a key role in the utilization of carbon sources, especially in the use of nonfermentable carbon sources.

Deletion of *Aosnf1*, *Aogal83*, and *Aosnf4* resulted in a remarkable reduction in conidia yield compared with the WT strain, which was consistent with previous reports in filamentous fungi, including Gibberella zeae (33), M. oryzae (10), Beauveria bassiana (34, 35), Pestalotiopsis microspore (36), and A. alternata (12). Furthermore, the spore morphology of A. oligospora, B. bassiana, and A. alternata exhibited no change, whereas the spores of G. zeae became shorter, and spores of M. oryzae were abnormal. In addition, compared with the WT strain, the transcription levels of several sporulation-related genes were varied in the AMPK mutants; for example, the transcription of *abaA* was downregulated in three mutants during the middle stage of conidiation (fifth day), whereas the *medA* was upregulated in mutants (Δ*Aosnf1* and Δ*Aosnf4*). In particular, *abaA*, along with *brlA* and *wetA*, forms a central regulatory pathway governing sporulation in filamentous fungi and regulates the transcription of genes with important developmental functions (36). In A. nidulans, deletion of *abaA* and *medA* resulted in abnormal conidial morphology (37–39). In F. graminearum, *abaA* deletion mutants completely abolished sporulation (40). These results suggest that AMPK plays a conserved role in the spore production of diverse filamentous fungi.

Previous studies have shown that AMPK protein kinase plays an important role in the pathogenicity of different fungi. In M. oryzae, the disruption of *Mosnf1*, *Mosip2*, and *Mosnf4* genes reduced the virulence of fungi, especially Δ*Mosnf1*, which almost completely lost virulence (10). In Metarhizium acridum, the pathogenicity of Δ*Masnf1* was decreased, which may be due to the reduction in appressorium formation and the decline in growth rate in insect hemolymph (41). In Penicillium digitatum, deletion of the *Pdsnf1* gene reduced the virulence of the mutant strain and the expression of many genes related to cell wall-degrading enzymes (42). The formation of traps is crucial for NT fungi to capture and kill nematodes (21). In this study, traps produced by the AMPK mutants were remarkably reduced, and more extracellular enzymes were detected in the Δ*Aosnf1* mutant than the WT strain. In addition, the nematode predatory efficiency was decreased in the Δ*Aogal83* and Δ*Aosnf4* mutants at 24 h and 36 h, whereas the nematocidal activity of the Δ*Aosnf1* mutant was decreased at 24 h, exhibiting no difference from the WT strain at 36 h. Previous studies have proven that extracellular serine protease is involved in the nematode predation of NT fungi by degrading the proteinaceous components of the nematode cuticle (43, 44). The increased proteolytic activity of Δ*Aosnf1* mutant may have resulted in the increase in predatory efficiency at 36 h. This was further confirmed by the variation of the transcription level of the genes encoding serine proteases in mutants compared with the WT strain. Thus, AMPK is involved in pathogenicity by regulating the development of infectious structures in A. oligospora and other pathogenic fungi, and *Aosnf1* may also participate in the regulation of production of serine proteases.

In yeast, oxidative stress resulted in the phosphorylation of Thr-210 and an increase in the catalytic activity of SNF1, and upstream kinases were required to phosphorylate Thr-210 to resist hypertonic stress (1 M NaCl) (45). Similarly in Pestalotiopsis microspora, the vegetative growth of the Δ*snf1* mutant was markedly inhibited under 1 M NaCl, 1 M sorbitol, 2 mM H_2_O_2_, and 0.02% Congo red compared to the controls (36). In A. alternata, the Δ*Aasnf1* mutant had reduced resistance to cell wall inhibitors SDS and Congo red, whereas there was no obvious difference in the growth rate between the WT and mutant strains supplemented with sorbitol, KCl, NaCl, and H_2_O_2_ (12). In this study, the Δ*Aosnf1* mutant strain had reduced resistance to oxidative stress (H_2_O_2_ and menadione) compared to the WT strain, and the Δ*Aogal83* and Δ*Aosnf4* mutants also had increased sensitivity to menadione. Accordingly, the downregulated transcription levels of most genes related to oxidative stress response were associated with their increased sensitivity to oxidative stress. In addition, the AMPK mutants had increased resistance to cell wall inhibitors (0.06 or 0.09 mg/mL Congo red), whereas the Δ*Aogal83* and Δ*Aosnf4* mutants showed increased sensitivity to osmotic stress (NaCl and sorbitol). These results indicate that AMPK is involved in the stress response, but its role may vary in different fungi.

Transcriptome analysis of the Δ*Aosnf1* mutant strain showed that upregulated DEGs were remarkably enriched into the KEGG pathways of peroxisome and fatty acid degradation during vegetative growth (0 h) and trap formation (12 h). Fatty acid degradation in most organisms occurs primarily via the β-oxidation cycle; β-oxidation occurs in both mitochondria and peroxisomes in mammals, whereas only the peroxisomes harbor the β-oxidation cycle in plants and most fungi (46). In fungi, the peroxisome plays an important regulatory role in the metabolism of nonfermentable compounds such as glycerol and fatty acids (47). In this study, the transcription of four enzymes involved in fatty-acid β-oxidation, namely, carnitine *O*-acetyltransferase (AOL_s00215g212), long-chain acyl-CoA synthetase (AOL_s00215g200), enoyl-CoA hydratase (AOL_s00215g56), and 3-ketoacyl-CoA ketothiolase (AOL_s00210g122), was upregulated. Meanwhile, fatty acid oxygenase PpoC (AOL_s00215g31) was also upregulated in the Δ*Aosnf1* mutant. It has been reported that PpoC is involved in the growth and development of fungi. In Aspergillus fumigatus, the Δ*ppoC* mutant produced fewer conidia, which were oval-shaped and larger, germinated faster in shaking cultures and slower in stationary cultures, and showed increased survival at both room temperature and 37°C, than the WT strain. In particular, the deletion of *ppoC* increased phagocytosis and killing by alveolar macrophages (48). Similarly, in A. nidulans, deletion of *ppoC* reduced conidia, increased ascospore production, and delayed conidiophore formation. PpoC was also shown to be involved in the production of lipogenic signal molecule psiB oxylipins (49). Recently, two peroxisome biogenesis genes, *pex1* and *pex6*, were characterized in A. oligospora; deletion of gene *pex1* or *pex6* resulted in a failure to produce traps and conidia, as well as a reduction in nematode predation (50). In M. oryzae, deletion of the *snf1* gene resulted in the absence of peroxisome structure, and mutant strains could not survive on medium with nonfermentable carbon sources (10). In fact, in the present study, the AMPK mutants showed a reduction in glycerol utilization capacity, LD accumulation, and conidial yield.

In addition, fatty acids can serve as the sole sources of carbon and energy; acetyl-CoA must be converted to C_4_ compounds via the glyoxalate bypass, comprising the enzymes isocitrate lyase and malate synthase, allowing gluconeogenesis (51). In this study, glyoxylate and dicarboxylate metabolism was downregulated in the Δ*Aosnf1* mutant at 0 h. Previous studies have proven that deletion of *Aomls* (encoding malate synthase) resulted in a reduction in conidiation and trap formation, as well as failure to utilize fatty acids and sodium acetate for growth; furthermore, its conidia were unable to germinate on minimal medium supplemented with sodium oleate (52). In Candida albicans, deletion of *Caicl1* (encoding isocitrate lyase) resulted in a failure to grow on the medium supplemented with citrate or glycerol as the sole carbon source, and the *icl1* gene was required for the full virulence of this fungal pathogen (53). Furthermore, the glyoxylate cycle is essential for the pathogenicity of bacterial pathogens, such as Mycobacterium tuberculosis, Rhodococcus equi, and Pseudomonas aeruginosa (53). Here, deletion of *Aosnf1*, *Aogal83*, and *Aosnf4* genes resulted in reduced glycerol utilization capacity and trap formation, as well as decreased pathogenicity at 24 h, consistent with the downregulated nitrogen metabolism, regulation of energy, and reduced glyoxylate and dicarboxylate metabolism. In addition, the downregulated terms of response to nutrient and oxidative stress were likely consistent with the mycelial growth and stress response of the mutants.

SNF1 plays a major role in lipid biosynthesis in S. cerevisiae, with deletion of *snf1* increasing lipid production (54, 55). Similarly, in Yarrowia lipolytica, the Δ*snf1* mutant accumulated more fatty acids than the WT strain (55). In Mortierella alpina, knockout of *Masnf4* contributed to fatty acid synthesis, whereas *Masip2* overexpression increased lipid production, and knockout of *Masip2* increased fatty acid unsaturation (56, 57). In our study, the LD volume of the Δ*Aosnf1*, Δ*Aogal83*, and Δ*Aosnf4* mutants was decreased compared with the WT strain, consistent with the expression of genes involved in lipid metabolism. In addition, more vacuoles and LDs were observed in the Δ*Aosnf1* mutant than the WT strain by transmission electron microscopy (TEM). Moreover, endocytic vesicles were observed in the WT strain but not in Δ*Aosnf1*; consequently, the associated downregulated endocytosis pathway was enriched in Δ*Aosnf1*. Taken together, our findings suggest that AMPK may play different roles in the lipid metabolism of different fungi, and it may affect the biosynthesis of cellular components.

**FIG 8:**
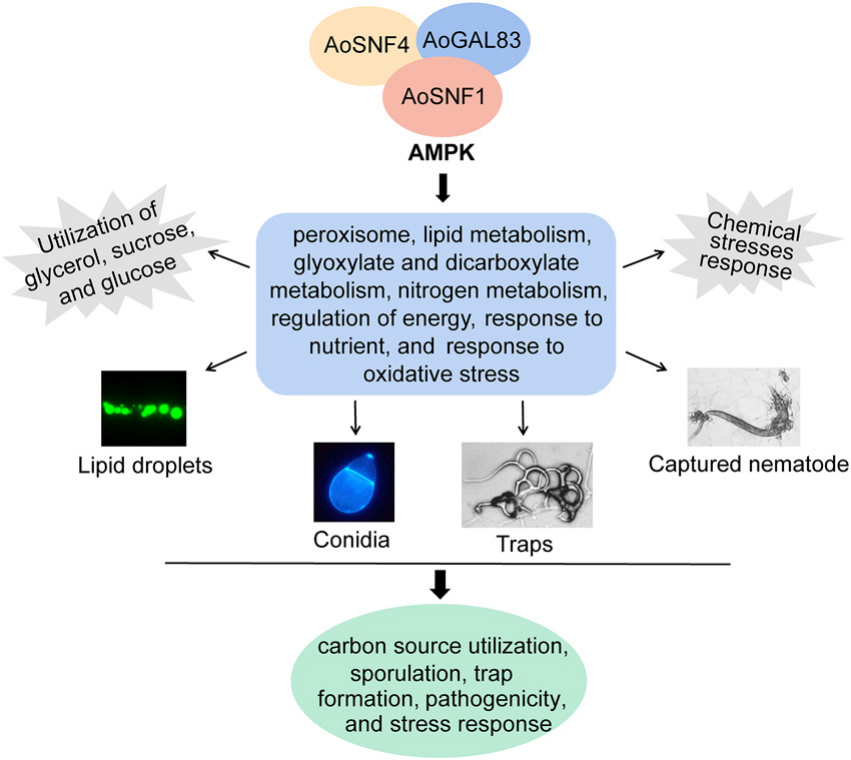
Schematic diagram of AMPK function regulation in A. oligospora. AMPK is involved in the peroxisome, lipid metabolism, glyoxylate and dicarboxylate metabolism, regulation of energy, nitrogen metabolism, response to nutrient, and response to oxidative stress, resulting in the regulation of carbon source utilization, sporulation, trap formation, pathogenicity, and stress response.

To sum up, we constructed the Δ*Aosnf1*, Δ*Aogal83*, and Δ*Aosnf4* mutant strains for the first time, and our results showed that three subunits of AMPK play a similar role in phenotypic traits of A. oligospora. A combination of phenotypic and transcriptome analyses between the WT and mutant strains suggested that AMPK is involved in the peroxisome, lipid metabolism, glyoxylate and dicarboxylate metabolism, regulation of energy, nitrogen metabolism, response to nutrients, and response to oxidative stress, ultimately regulating the carbon source utilization, sporulation, trap formation, pathogenicity, and stress response in A. oligospora (Fig. 8). It is worth noting that the trap formation process requires energy (20), whereas AMPK can sense cellular energy and maintain cellular energy homeostasis (2). Deletion of *Aosnf1*, *Aogal83*, and *Aosnf4* genes resulted in a significant reduction in the traps of A. oligospora. Therefore, further exploring the mechanisms underlying how AMPK influences trap formation and development through energy regulation will provide new insights into the lifestyle switching of NT fungi.

## MATERIALS AND METHODS

### Strains and culture conditions.

The WT strain A. oligospora (ATCC 24927) and the mutant strains Δ*Aosnf1*, Δ*Aogal83*, and Δ*Aosnf4* were cultivated on PDA at 28°C. S. cerevisiae (FY834) was cultured in yeast extract-peptone-dextrose (YPD) medium and inoculated on synthetic complete medium lacking uracil (SC-Ura) for screening the recombinational clones (58). The Escherichia coli strain DH5α (TaKaRa, Shiga, Japan) was maintained on Luria-Bertani (LB) medium for storing plasmids pCSN44 and PRS426. PDA, TG, water agar (WA), and cornmeal-molasses-yeast (CMY) medium were used for various phenotypic analyses, and Czapek-Dox agar medium was used to compare the utilization of carbon sources between the WT and mutant strains (12). Caenorhabditis elegans (strain N2) was incubated at 26°C on oatmeal medium for trap induction of A. oligospora.

### Sequence and phylogenetic analyses of AMPK complex in A. oligospora.

The sequences of SNF1 (AOL_s00173g192), GAL83 (AOL_s00215g614), and SNF4 (AOL_s00004g493) were retrieved from A. oligospora using the homologous sequences of S. cerevisiae as a query. The NCBI database was used to search the homologous sequences of the A. oligospora AMPK subunits in different fungi. A neighbor-joining tree was constructed using MEGA 5.0 software (59). The InterProScan database was used to predict and analyze the conserved domains and functional sites (60).

### Deletion of *Aosnf1*, *Aogal83*, and *Aosnf4* genes.

The *Aosnf1*, *Aogal83*, and *Aosnf4* genes were deleted by homologous recombination as described previously (61). The 5′ and 3′ flanking sequences of the target genes and the hygromycin resistance gene cassette (*hph*) were amplified from A. oligospora and pCSN44 using primers (see Table S2 in the supplemental material), respectively. These three DNA fragments and linearized pRS426 vector were cotransformed into S. cerevisiae FY834 by use of electroporation. Then, the complete fragments for targeted gene disruption were individually transformed into A. oligospora protoplasts (62). For selection of positive transformants, PDA supplemented with 0.6 M sucrose (PDAS) plates supplemented with 200 mg · mL^−1^ hygromycin were used, and PCR and Southern blot analyses were used to further substantiate them (Fig. S1).

### Comparison of mycelial growth, utilization of carbon sources, and conidiation.

The WT and mutant (Δ*Aosnf1*, Δ*Aogal83*, and Δ*Aosnf4*) strains were inoculated on PDA and TG plates at 28°C for 6 days, respectively, and we recorded colony growth and measured colony diameter. By use of Czapek-Dox agar medium as the base medium, various carbon sources (glycerol, sucrose, and glucose) were added or not to test the carbon source utilization capacity of the WT and mutant strains, and the colony diameter was measured after 6 days of incubation (12). After 14 days of incubation on CMY, 20 mL of sterile double-distilled water (ddH_2_O) was added to elute and collect the spore suspension, followed by counting under a microscope (63). The spores were stained with 20 μg/mL calcofluor white (CFW; Sigma-Aldrich), and the morphology of the conidia was visualized under a light microscope (64).

### Trap development and pathogenicity assays.

The above-described spore suspension was spread on the WA plates (2 × 10^4^ conidia/plate) and cultured at 28°C for 3 to 4 days. Caenorhabditis elegans (cultured on oatmeal medium) was washed with sterile ddH_2_O, and then about 400 nematodes were added to the WA plates (64). At 12, 24, 36, and 48 h, the number of traps and captured nematodes were counted, and the percentages of captured nematodes of the WT and mutant strains were calculated.

### Extracellular protease activity analysis.

Skim milk was added to the PDA medium, and then the fungal strains were inoculated for 7 days at 180 rpm at 28°C. The fermentation broth was collected, and the extracellular enzyme activity was determined on the skim-milk plates as previously described (22).

### Stress response.

Using TG solid medium, the response of fungal strains to environmental stress was detected. The WT and mutant strains were inoculated in TG medium containing osmotic stressors (sorbitol and NaCl), cell wall-perturbing agents (Congo red and SDS), and oxidative stressors (H_2_O_2_ and menadione) at 28°C for 6 days. Different concentrations of chemical stressors were added as described in previous reports (26).

### Transcriptome and lipid staining analysis.

The WT and Δ*Aosnf1* mutant strains were incubated onto PDA plates with cellophane on the surface at 28°C. After 3 days of incubation, equal amounts of nematodes were added for induction for 0 and 12 h, and mycelia were collected. Three independent biological replicates were used for each sample, and hyphal samples (12 samples) were sent to the Shanghai Meiji Biological Company (Shanghai, China) for RNA sequencing and analysis (65). GO and KEGG analyses were performed using the free online Majorbio Cloud Platform (http://www.majorbio.com).

Lipid droplets were stained with 10 mg/mL BODIPY (Sigma-Aldrich, St. Louis, MO, USA) for 30 min and observed under a confocal laser scanning microscope.

### RT-PCR analysis.

The WT and mutants were inoculated on CMY or PDA plates at 28°C, and mycelia were collected at different time points. The total RNA of mycelial samples was extracted and reverse transcribed to cDNA as previously described (64). Primer pairs for the target genes related to conidiation, oxidative stress response, serine proteases, peroxisome, glycerolipid metabolism, and endocytosis were designed (Table S3); the transcriptional levels of genes were detected by the LightCycler 480 SYBR green I master mix (Applied Biosystems, Germany) using cDNA as a template (63). The β-tubulin gene was used as an internal standard. The relative transcription level of each gene was calculated using the threshold cycle (2^−ΔΔ^*^CT^*) method (66).

### Statistical analysis.

All experiments were biologically replicated three times, and the data were expressed as mean values ± standard deviations (SD). Statistical analysis was performed using Prism 8 (GraphPad Software, San Diego, CA, USD), and Tukey’s honestly significant difference (HSD) test was used to analyze the significance of the differences among treatments.

### Data availability.

All data generated or analyzed during this study are included in the published paper and the associated supplementary files. The raw sequence was deposited to the GEO under accession number GSE205188.
