# Supplementary material for: AMPK Is Involved in Regulating the Utilization of Carbon Sources, Conidiation, Pathogenicity, and Stress Response of the Nematode-Trapping Fungus Arthrobotrys oligospora
Source: Microbiol Spectr. 2022 Aug 2;10(4):e02225-22. doi: 10.1128/spectrum.02225-22 (PMC9431048; doi:10.1128/spectrum.02225-22)
Supplement: Supplemental file 1 — Supplemental material. Download spectrum.02225-22-s0001.pdf, PDF file, 2.7 MB [file spectrum.02225-22-s0001.pdf]

SUPPLEMENTARY MATERIALS

Supplementary Figures

Figure S1. Deletion and verification of *Aosnf1*, *Aogal83*, and *Aosnf4* genes in *A. oligospora*. (A) Schematic diagram of homologous recombination of *Aosnf1*, *Aogal83*, and *Aosnf4* and the homologous flanks of the target genes. (B) PCR validation of five-generation of AMPK mutants. (C) Southern blot verification of the wild-type (WT) strain and transformants. Two independent transformants for each gene was verified.

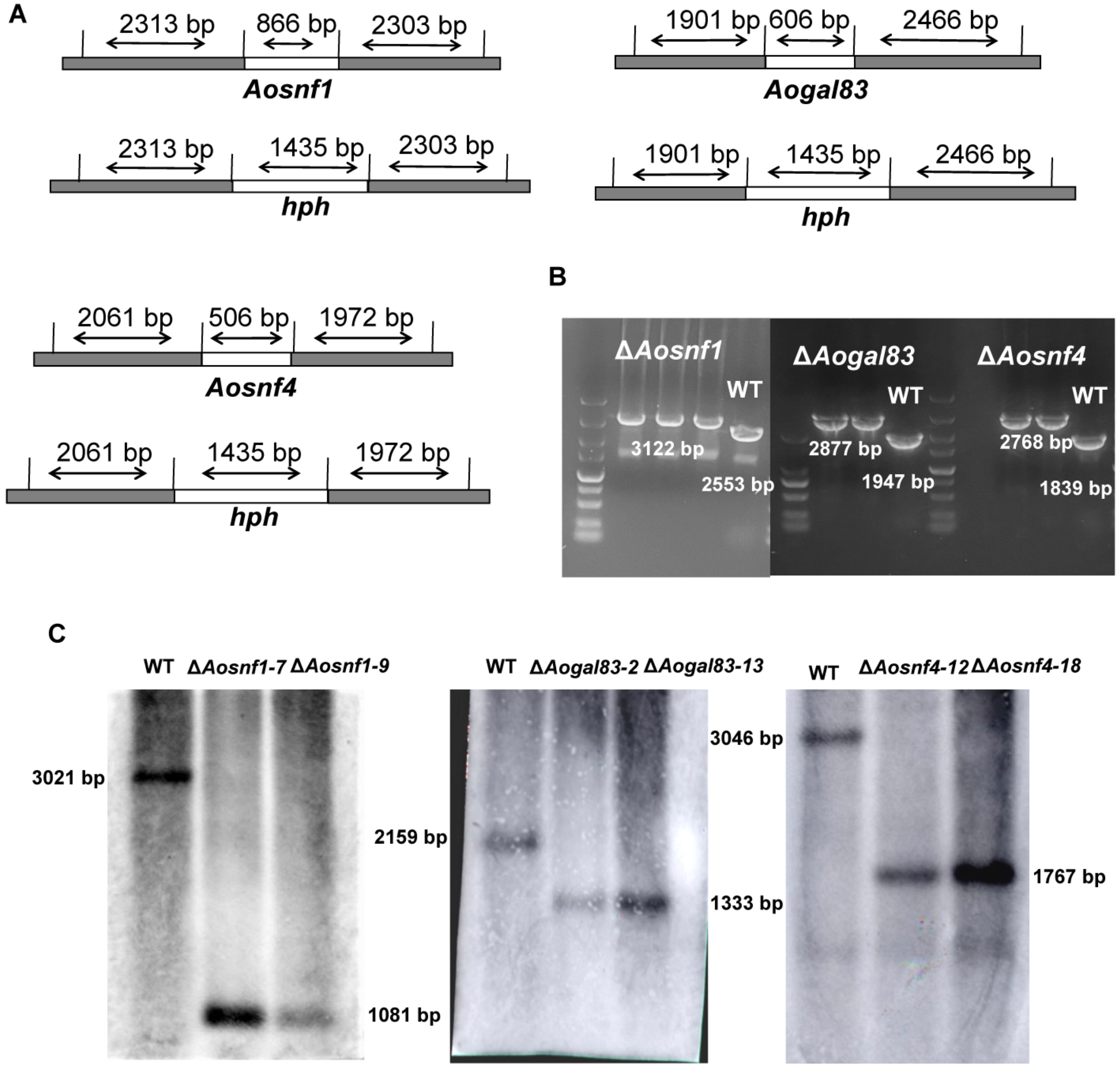

Figure S2. Comparison of stress response between the WT and mutant strains. (A and B) Growth and relative growth inhibition (RGI) values of the WT and Δ*Aosnf1* mutant strains on TG media supplemented with 0.09 mg/mL Congo red and 0.2 M NaCl. (C and D) Growth and RGI values of the WT and Δ*Aogal83* mutant strains on TG media supplemented with 0.06 mg/mL Congo red, 0.02 % SDS, 0.5 and 0.75 M sorbitol. (E and F) Growth and RGI values of the WT and Δ*Aosnf4* mutant strains on TG media supplemented with 0.06 and 0.09 mg/mL Congo red, 0.3 M NaCl, and 0.25 M sorbitol. The asterisk (B, D, and F) indicates the significant difference between the mutant and WT strains (Tukey's HSD, $p < 0.05$).

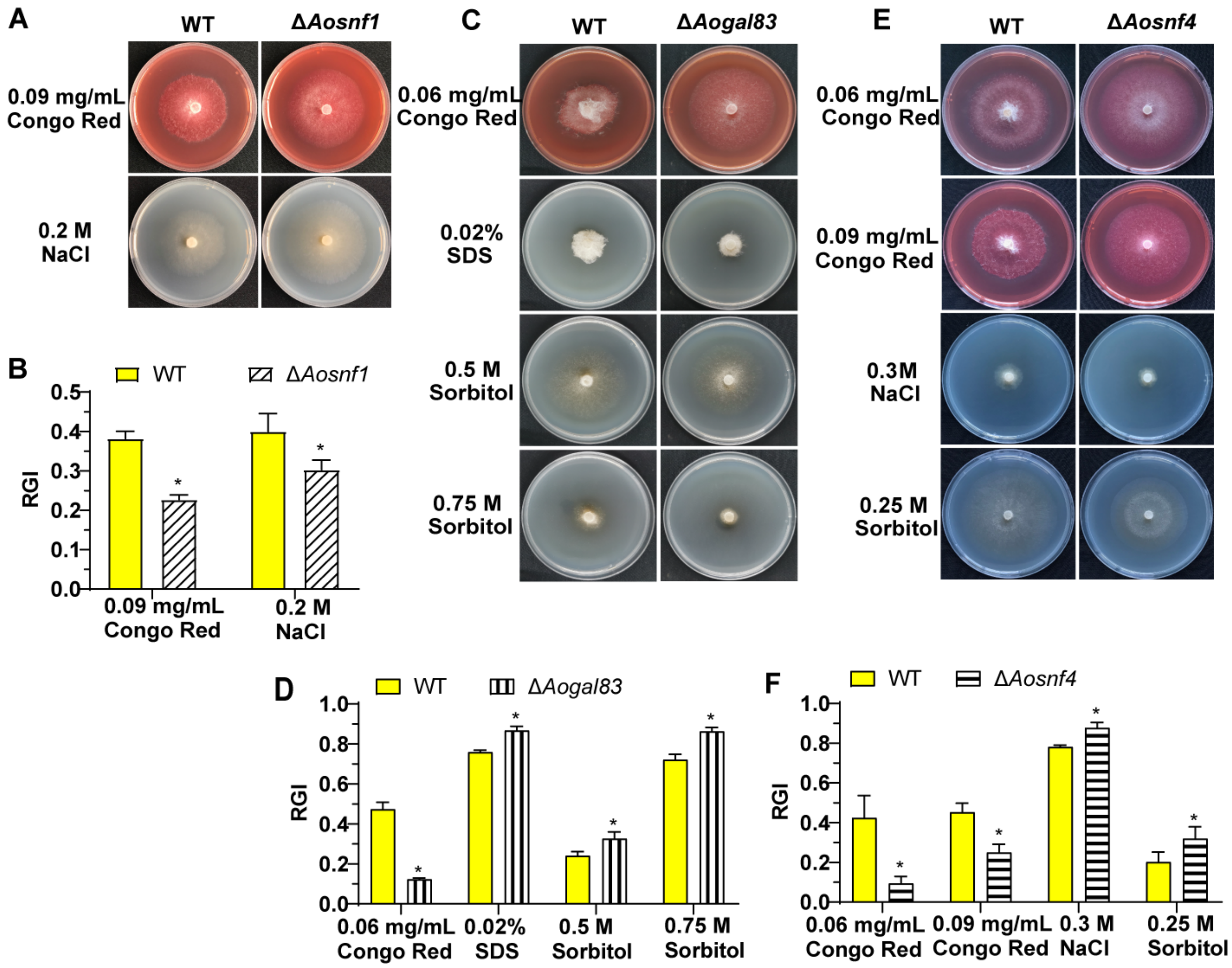

Figure S3. PCA analysis among samples. A1 and A2 represent the WT samples induced by nematodes at 0 and 12 h, respectively. B1 and B2 represent the Δ*Aosnf1* mutant samples after nematode induction for 0 and 12 h, respectively.

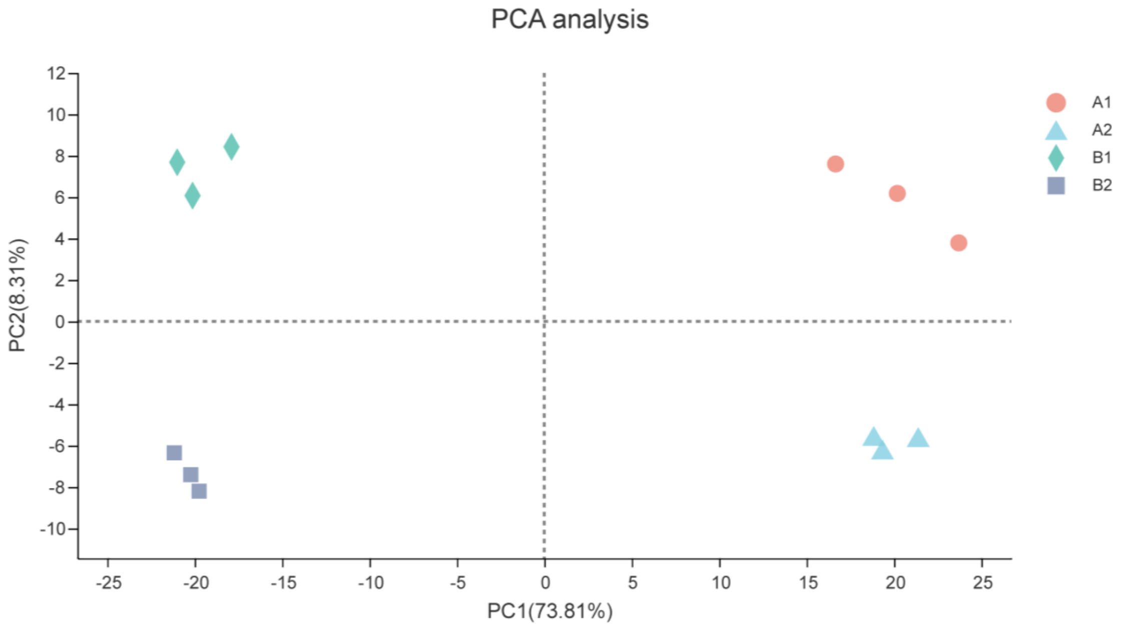

Figure S4. Reverse Transcription-Polymerase Chain Reaction validation of transcriptome data in the Δ*Aosnf1* mutant vs the WT strain at 0 and 12 h. *, significant difference between the mutant and WT strains ($p < 0.05$). CK is the standard (RTL = 1) for statistical analysis of the RTL of each gene under a given condition.

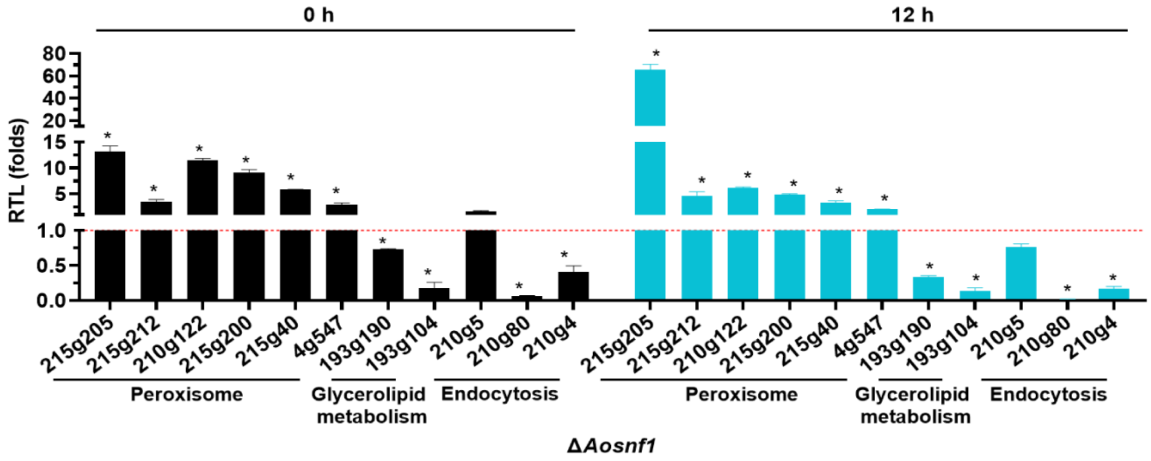

Figure S5. Gene Ontology (GO) enrichment analysis. (A) The number of GO terms obtained after GO enrichment analysis. (B, C, D, and E) GO enrichment analysis of upregulated and downregulated genes in the Δ*Aosnf1* mutant vs the WT strain at 0 and 12 h, respectively.

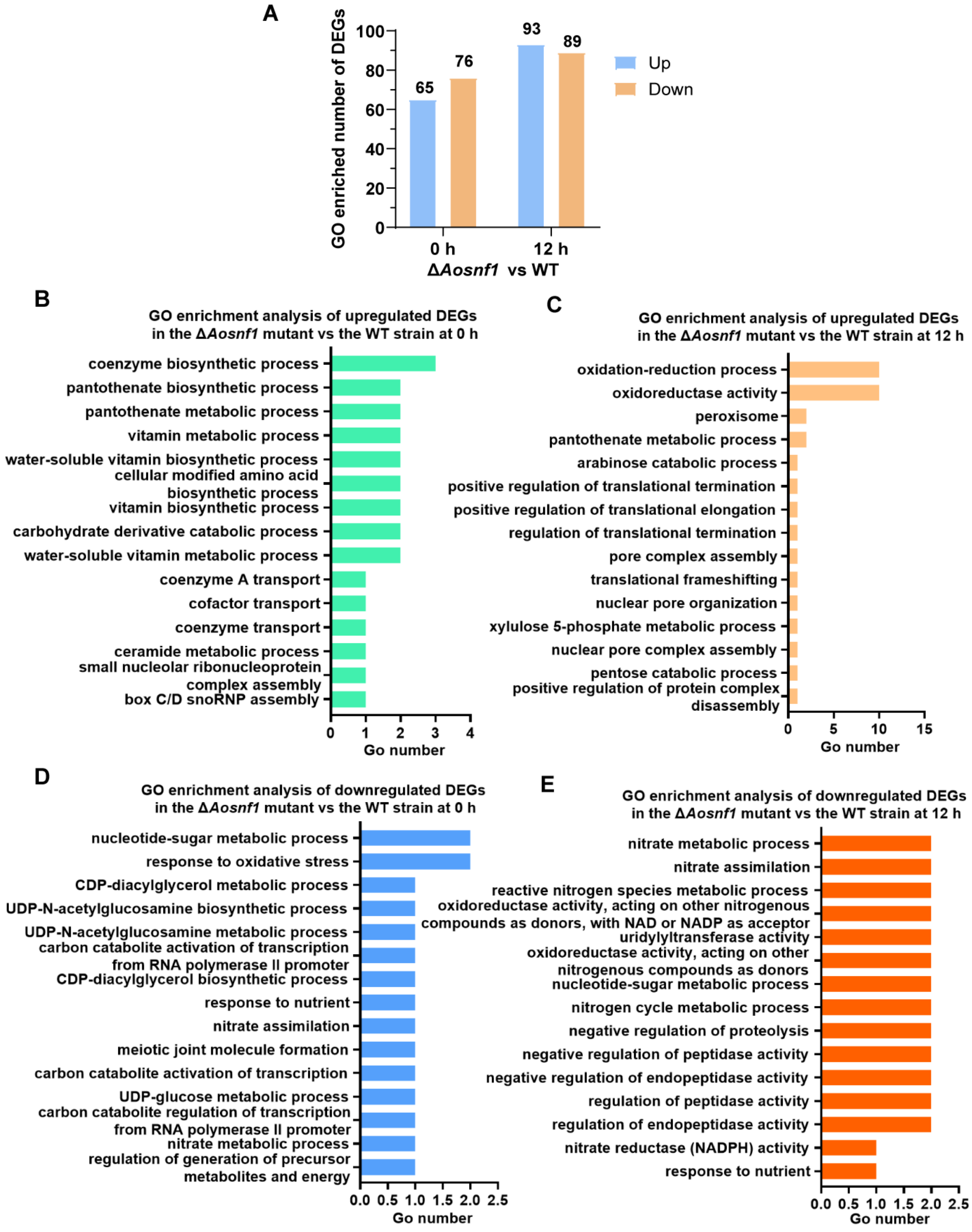

Figure S6. KEGG analysis of upregulated and downregulated genes in the Δ*Aosnf1* mutant vs the WT strain at 0 h.

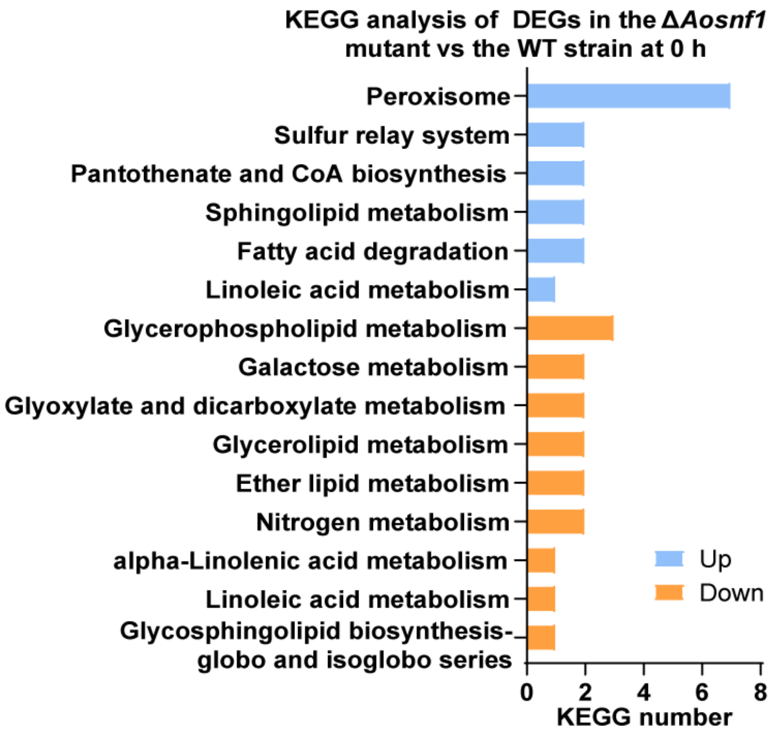

Supplementary tables

Table S1. Quality control data statistics of 12 samples.

Sample	Clean reads	Clean bases	Error rate (%)	Q20 (%)	Q30 (%)	GC content (%)
A_0_1	42560316	6336672650	0.0252	97.94	93.9	48.24
A_0_2	44723046	6663963052	0.0253	97.89	93.85	48.23
A_0_3	41418288	6176756195	0.025	98.03	94.19	48.44
A_12_1	46595278	6933776938	0.0252	97.95	93.96	48.3
A_12_2	40937466	6093238748	0.0255	97.8	93.65	48.4
A_12_3	41521656	6195039571	0.0249	98.05	94.23	48.5
B_0_1	44069738	6562631377	0.0249	98.05	94.2	48.29
B_0_2	44179466	6595686792	0.0248	98.1	94.34	48.58
B_0_3	47789858	7127226525	0.0249	98.06	94.24	48.68
B_12_1	50157288	7437404739	0.0252	97.94	93.95	48.8
B_12_2	45126770	6724261008	0.025	98.04	94.16	48.82
B_12_3	46408086	6873713782	0.0246	98.18	94.55	48.79

Table S2. Paired primers for genes disruption in this study.

Primers	Sequence (5′-3′)	Description
hphF	GTCGGAGACAGAAGATGATATTGAAGGAGC	Amplify the *hph* cassette
hphR	GTTGGAGATTTCAGTAACGTTAAGTGGAT	
173g192-5f	CACCACGGTAAAGGGCATAG	Amplify *Aosnf1* gene 5’ flank
173g192-5r	GAAATGGCAGTTCGGTAT	
173g192-3f	TTCTGTTGACAGGGTTGA	Amplify *Aosnf1* gene 3’ flank
173g192-3r	GACCTATTACGAATTACCG	
215g614-5f	CTGTTTTGTCTGACGGTGCG	Amplify *Aogal83* gene 5’ flank
215g614-5r	GCCTCCTTCGGTCCATTCAA	
215g614-3f	GCTCCTCATCATTCACCCGT	Amplify *Aogal83* gene 3’ flank
215g614-3r	GTGCGTTCTATTTGACCGGC	
4g493-5f	GCCTTACATTCATTTGGA	Amplify *Aosnf4* gene 5’ flank
4g493-5r	ATTCATCTGCTGGTCAAA	
4g493-3f	GTGCTGAAACAATGCCTAT	Amplify *Aosnf4* gene 3’ flank
4g493-3r	ACAGTACGACCGCAAAGA	
YZ-173g192-5f	CTTCCGTTCATTCTCCTT	Verify the transformants
YZ-173g192-3r	ACGACCACCTACCTTCTT	
YZ-215g614-5f	CCCACCATCACCACTTCC	
YZ-215g614-3r	CGACAGAGCCAGCAAACC	
YZ-4g493-5f	CGAAGAAGCGATGAGGAA	
YZ-4g493-3r	GTGGTGGAAGAAGAACAGAA	
TZ-173g192-5f	CCTCAGGTCCAGCATACA	Make Southern blot probes
TZ-173g192-3r	GTTACGGCATCCTCACAT	
TZ-215g614-5f	CGATTGAGCGTCTACTTT	
TZ-215g614-3r	CATACACCGGGAGAAATA	
TZ-4g493-5f	AGTTATCGTTGGCATCCT	
TZ-4g493-3r	ATCACTAAACTCCGCTCTT	

Table S3. List of primers for reverse transcription-polymerase chain reaction in this study.

Sporulation-related genes	Sequence (5′-3′)
AOL_s00169g18 (*veA*)	18-5F- AAGCTACACCCAATCAACGC
	18-3R- TTGCGATGCTGACGATCTTG
AOL_s00043g361 (*fluG*)	361-5F-GATTCCAGTCCCGTGAATTC
	361-3R-GCTAAGGAGAGGATGGGCAT
AOL_s00080g63 (*abaA*)	63-5F-AACTTTATGCGCCTTGTCGT
	63-3R-TTGGCTAGGTGGTCTGTACG
AOL_s00210g120 (*medA*)	120-5F-TCCGGCCCAATGATTCAGAA
	120-3R-AGATCGCAGGAACATGGTGA
AOL_s00083g487 (*lreA*)	487-5F-TTCTCTTCGTCCCAAGCCAC
	487-3R-ACCGGTTCGAGTGGAGTCTA
AOL_s00007g157 (*flbC*)	157-5F-CTCTCCGGCAAAGACAATCG
	157-3R-GTCGACTGAGGATAGTAGCT
AOL_s00054g700 (*vosA*)	700-5F-CAAACCACCCACCACCAAAT
	700-3R-GGATGGACAGGAGAAGGACC
AOL_s00215g516 (*flbA*)	516-5F-TTCAAACGCAGCTCCTTCAC
	516-5R-AAGCGGGTTGACAGATGAGA
**Genes encoding serine proteases**	**Sequence (5′-3′)**
AOL_s00076g4	4-5F-CCATGGTGTTGGAAGGAAAT
	4-3R-GTCTGTTCAGCGTACGTAGT
AOL_s00075g8	8-5F-TTGCTACTTTACTGCCCCTTG
	8-3R-TCTTCAGCTTGAGTCCGGTT
AOL_s00112g42	42-5F-CTGGCTCTTGGCCTACTTTG
	42-3R-AGGAGGTTGACGGTCTCCTT
AOL_s00078g136	136-5F-ACACTTGCCCATTTCACTCC
	136-3R-GCTGGGTTTCACAACATCCT
AOL_s00215g702	702-5F-GTCGCCGCTGACTTAACTGT
	702-3R-ATAATTGCTGATTCGCTGGG
AOL_s00054g992	992-5F-TCCGCAACTTCAAGAGTGTG
	992-5R-CGTTGGCTTCCTCGTTAGAG
**Genes related to oxidative stress response**	**Sequence (5′-3′)**
AOL_s00054g13 (*glr*)	13-5F-CGAAAAGTCTGAATCGGGTGA
	13-3R-GCTCCACTTTGCCACATACATC
AOL_s00043g396 (*gld*)	396-5F-TTTGGATGTGGTGCTGTTGG

	396-3R-CTGTCGCCTCAATGATCGTG
AOL_s00215g326 (*per*)	326-5F-CACCATCCGCTCTGTCTTCA
	326-3R-GACGGCATCCTCGGTCTTGA
AOL_s00054g257 (*thi*)	257-5F-CCAAGACGAAAACGGACAAG
	257-3R-CCTGGAAGGGTGCAATAACT
AOL_s00173g374 (*cat*)	374-5F-CCGCCAAGTTCCCACATT
	374-3R-GCTTCTGGGTTTTGGCTCA
**Genes related to peroxisome**	**Sequence (5′-3′)**
AOL_s00215g205	205-5F-CAATACTCGCATGACAGCTC
	205-3R-TTTCTTCTTCTCAACAAAGT
AOL_s00215g212	212-5F-GATTTGATCAACCAGAACGA
	212-3R-AGTGCATCGATCTTAGCCTT
AOL_s00210g122	122-5F-GCCGCACATATTGTTAACAGAT
	122-3R-TGATCTTGCTGTTCTCAGTCAT
AOL_s00215g200	200-5F-GATATGGACAAGGTCGCTAAGA
	200-3R-CTTGGCGTTAATAGGTGATTCG
AOL_s00215g40	40-5F-CTCGACTCCTCGGGTATTTTTA
	40-3R-GAGAAAACAAGTCCGGTAAACC
AOL_s00004g547	547-5F-GAGTACCATATCTCAAGCGGAA
	547-3R-CAGCATTGATCACTGGATATGC
**Genes related to glycerolipid metabolism**	**Sequence (5′-3′)**
AOL_s00193g190	190-5F-AGGAGAGGCATTTTTCGTTTTC
	190-3R-GTTTCCAAATCTAGCACGGATC
AOL_s00193g104	104-5F-CGTCTTCATGCTAAACACTCTG
	104-3R-GTGTGTTCTTTTTCCCTTACCC
**Genes related to endocytosis**	**Sequence (5′-3′)**
AOL_s00210g5	5-5F-TACTCCTCCTGTTGTAAAGCAG
	5-3R-TTCACGATGCCATTGCTATTAC
AOL_s00210g80	80-5F-GTGAAACACCACTATCGTATGC
	80-3R-CTTAGCCTCCAAGTTTGCTTTT
AOL_s00210g4	4-5F-AATGTCGACAAACAACATCTCG
	4-3R-CTCTGTTGTTGAACATTGAGCA
